# Comparative Assessment of Fermented and Non-Fermented Berry Seeds as Sources of Functional Oils

**DOI:** 10.3390/foods14203494

**Published:** 2025-10-14

**Authors:** Audrone Ispiryan, Elvyra Jarienė

**Affiliations:** Bioeconomy Research Institute, Agriculture Academy, Vytautas Magnus University, Studentu Str. 11, LT-53361 Akademija, Lithuania; elvyra.jariene@vdu.lt

**Keywords:** berry seeds, cold-pressed oils, fermentation, fatty acid composition, tocopherols, phenolics, antioxidant activity, oxidative stability, valorization

## Abstract

Berry seeds represent an underexploited byproduct of juice and wine production, and are increasingly valued sources of high-quality cold-pressed oils. In this study, eight berry species, including blackcurrant (*Ribes nigrum*), red currant (*Ribes rubrum*), raspberry (*Rubus idaeus*), strawberry (*Fragaria*), sea buckthorn (*Hippophae rhamnoides*), honeysuckle (*Lonicera caerulea*), viburnum (*Viburnum opulus*), and rowanberry (*Sorbus aucuparia*), were investigated to determine the impact of primary fermentation on seed composition and oil quality. Seeds obtained from juice production were compared with those obtained after fermentation. Fermentation consistently reduced seed sugars and carbohydrates by more than 50% while increasing relative protein levels, demonstrating microbial utilization of fermentable substrates. Oil yields showed species-specific responses, with blackcurrant and honeysuckle seeds increasing from ~14 to 15% and ~7 to 8%, respectively, while raspberry decreased from ~9 to 8%, and viburnum decreased from ~6 to 5%. Importantly, fatty acid profiles remained unchanged across all treatments, confirming that fermentation does not alter the natural dominance of linoleic and α-linolenic acids. Tocopherol and total phenolic contents decreased modestly in fermented oils (typically 5–10%), which was reflected in small reductions of DPPH scavenging activity (2–4%) and oxidative stability (0.2–0.5 h). A multivariate heatmap and PCA analyses revealed that berry species identity was the primary driver of biochemical variation, while fermentation introduced only minor within-species shifts. The results indicate that berry pomace remaining after fermentation can still be valorized for cold-pressed oil production with minimal compromise in quality.

## 1. Introduction

Berry fruits such as blackcurrant *(Ribes nigrum*), red currant (*Ribes rubrum*), raspberry (*Rubus idaeus*), strawberry (*Fragaria*), sea buckthorn (*Hippophae rhamnoides*), blue honeysuckle (*Lonicera caerulea*), and viburnum (*Viburnum opulus*) are widely valued for their juices, jams, and wines [1,2,3]. Berry seeds are increasingly recognized as valuable byproducts of juice and wine production, owing to the high nutritional and functional quality of their oils. On a dry weight basis, berry seeds typically contain 10–30% oil [4,5,6,7]. Fatty acid composition is dominated by polyunsaturated fatty acids (PUFAs), with linoleic acid (C18:2, ω-6) accounting for 40–65%, α-linolenic acid (C18:3, ω-3) for 15–25%, and smaller but significant amounts of γ-linolenic acid (GLA, C18:3, ω-6) in *Ribes nigrum* (blackcurrant) oil (12–18%) [8,9]. In contrast, viburnum oil is distinguished by its high oleic acid content (50–60%), which sets it apart from other berry oils [10].

Beyond fatty acids, these oils are rich in bioactive compounds. Tocopherols are present at 200–400 mg/100 g of oil, with α-tocopherol being the predominant homologue. These molecules act as antioxidants, generally improving oxidative stability; however, at very high concentrations, tocopherols may also exert pro-oxidant effects under specific conditions. In addition, berry seeds retain phenolic compounds such as catechins, gallic acid derivatives, and flavonoids, with total phenolic contents reported in the range of 20–90 mg gallic acid equivalents (GAE)/100 g oil, depending on species and extraction method [11,12]. A portion of these phenolics is present in the seeds and can be co-extracted into the oil in small but significant quantities [13,14,15]. The combination of polyunsaturated fatty acids, tocopherols, and phenolics underpins the nutritional, functional, and oxidative properties of berry seed oils, contributing to their application in functional foods, health-related products, and cosmeceutical uses equally. Although tocopherols generally protect against oxidation, in high concentrations they may paradoxically accelerate pro-oxidant reactions under certain conditions.

Primary fermentation in winemaking or fruit fermentation is a critical context in which berry seeds undergo biochemical and physical changes. During alcoholic fermentation, crushed berry mash is inoculated with yeast to convert sugars into ethanol. The process generates heat and ethanol and lowers pH, thereby creating an environment that can affect the seeds embedded in the fermenting pomace. In classical grape winemaking, seeds remain in contact with fermenting must for days, allowing partial extraction of seed tannins and phenolics into the wine. By analogy, when berries like blackcurrants or raspberries are fermented, their seeds are exposed to yeasts and fermentation metabolites. Fermentation-induced cell wall degradation may increase the accessibility of seed components [16]. Studies have shown that fermenting plant matrices can liberate bound antioxidants and phenolics due to microbial enzymatic activity and acidic conditions [17,18,19,20]. In fermented berry pomace, for instance, the total phenolic content and antioxidant capacity can be enhanced relative to unfermented pomace, presumably as cell walls break down and phenolics become more extractable. On the other hand, fermentation may also cause leaching or biochemical transformation of seed constituents. Phenolic compounds, tocopherols, and vitamins may diffuse into the liquid phase or be metabolized by microorganisms. Additionally, prolonged contact with oxygen during pomace handling and the exothermic heat of fermentation may accelerate oxidative reactions in the seed oil [21,22]. Thus, it is plausible that fermentation influences both the yield and quality of the oil that is obtainable from seeds. However, fermentation could also negatively impact oil quality by reducing certain antioxidants or introducing volatile compounds.

Despite these considerations, a clear knowledge gap exists regarding how primary fermentation affects berry seed composition and oil quality. Most prior research has characterized berry seed oils from fresh seeds or industrial press cakes without fermentation. Little is known about whether “spent” fermented seeds produce oils of comparable nutritional and oxidative quality to oils from unfermented seeds [23]. This is an important question for the sustainable utilization of berry pomace. If fermentation diminishes oil yield or degrades sensitive compounds like polyunsaturated lipids or tocopherols, producers might need to adjust processing strategies. Conversely, if fermented seed oils retain high PUFA levels and antioxidant content, then fruit wine pomace could be an excellent feedstock for edible oils, adding value to what is otherwise waste. Grape seed oil production already exemplifies this concept. Grape seeds left over from wine making are routinely extracted to yield edible oil. For the diverse berries in question, like blackcurrant, red currant, raspberry, strawberry, sea buckthorn, honeysuckle, and viburnum, no comparative studies have yet elucidated the impact of fermentation on berry seed oil profiles [24]. Addressing this gap is crucial for developing integrated berry processing pipelines that maximize recovery of high-quality oils and bioactives from all stages.

Therefore, this study compared the composition and quality of oils from non-fermented (NF) and fermented (F) berry seeds by analyzing key parameters before and after primary fermentation to determine how fermentation alters seed nutritional properties. This knowledge will clarify whether fermented berry seeds can be as valuable as fresh seeds for oil production, providing a basis for the food, cosmetic, and nutraceutical industries to integrate berry pomace valorization into sustainable processing chains.

## 2. Research Methods

### 2.1. Sample Preparation

Seven berry species were used as the raw material: blackcurrant (*Ribes nigrum* L., variety “*Titania*”), strawberry (*Fragaria × ananassa* Duch., variety “*Florence*”), red currant (*Ribes rubrum* L., variety “*Jonkheer van Tets”*), raspberry (*Rubus idaeus* L., variety “*Polka*”), sea buckthorn (*Hippophae rhamnoides* L., variety “*Leikora*”), blue honeysuckle (*Lonicera caerulea* L., variety “*Wojtek*”), and viburnum (*Viburnum opulus* L., local landrace, Lithuania). All the berries were cultivated in the same geographic region (Central Lithuania, Kaunas District, latitude 54°53′ N, longitude 23°51′ E) under similar conditions. Harvesting took place from June to September 2024 during dry conditions; the berries were stored at −29 °C with controlled humidity and air circulation in a blast freezer (Liebherr, Ochsenhausen, Germany) and then processed as described below.

### 2.2. Juice-Making Procedure

For the juice residue (non-fermented) group, berries were pressed using a hydraulic juice press (Voran 180K, Voran Maschinen GmbH, Pichl bei Wels, Austria). Pomace consisting of skins and seeds was collected, dried, and used for seed separation. This served as the non-fermented control.

### 2.3. Primary Fermentation

For the fermented seed group, the berries were crushed to a pulp (including skins, pulp, and seeds) and prepared for primary fermentation. To the berry mash, two-thirds of the fruit mass of water and half of the fruit mass of sugar were added, and the mixture was thoroughly mixed, simulating fruit wine production conditions. Fermentation was carried out in food-grade fermentation vessels (Speidel 60 L, Speidel Tank-und Behälterbau GmbH, Ofterdingen, Germany) at 21 °C for 14 days. A commercial active dry yeast (Saccharomyces cerevisiae strain) Oenoferm^®^ Universal (Erbslöh, Geisenheim, Germany) was used as the starter culture to inoculate the mash. Yeast was added according to the manufacturer’s instructions (approximately 20–30 g per 100 L of mash). After inoculation, the mixture was stirred and sealed with an airlock, and every two days, the fermenting mash was thoroughly mixed to ensure uniform fermentation and prevent sedimentation. Fermentation progress was monitored daily by observing CO_2_ release and measuring density changes (measured with a DMA 35 portable densitometer, Anton Paar, GmbH, Graz, Austria). After 14 days, the process was considered complete, as indicated by the cessation of CO_2_ evolution and the stabilization of specific gravity.

### 2.4. Seed Separation and Drying

Two seed origin groups were distinguished for further analysis: the fermented seed group, obtained from fermented berry mash, and the juice residue seed group, obtained from pomace after juice production. After fermentation, the berry mash was skimmed and dried at 35 °C in a low-temperature infrared dryer (Infradry IRD, Kreyenborg GmbH, Senden, Germany) until the seed moisture content reached 9–12%, as confirmed using a moisture analyzer (measured using a MB90 moisture analyzer, OHAUS, Corporation, Parsippany, NJ, USA). Once dried, the material was sieved through a fine mesh (aperture 1–2 mm) to separate the seeds from berry skins and other residues. Dried seeds and press cakes were milled into fine powders using a laboratory mill (Retsch ZM200, Retsch GmbH, Haan, Germany, 0.5 mm sieve) prior to composition analysis.

### 2.5. Oil Extraction

Cold-press oil extraction was performed using a laboratory-scale cold press expeller (Komet CA59G, IBG Monforts Oekotec GmbH & Co. KG, Mönchengladbach, Germany). The cold pressing was performed without external heating, so that the oil temperature during extraction remained low (below 40 °C). Crude oils were filtered through cheesecloth and centrifuged (Hettich Rotina 420R, Tuttlingen, Germany) to remove solids. The oil was stored in amber glass bottles at −29 °C until analysis. Residual oil in press cakes was determined gravimetrically via Soxhlet extraction with n-hexane (AOCS Am 2-93). Results are expressed as % dry mass.

### 2.6. Fatty Acid Composition Analysis

The fatty acid composition of the seed oils was determined via gas chromatography (Shimadzu, Kyoto, Japan) with flame ionization detection (GC-FID). Prior to GC analysis, fatty acid methyl esters (FAMEs) were prepared from the oil samples. Approximately 50 mg of oil was subjected to transmethylation by adding 2 mL of 0.5 N methanolic KOH and heating at 60 °C for 10 min, followed by the addition of 2 mL hexane to extract the FAMEs (upper layer). After phase separation, the hexane layer containing FAMEs was collected for GC analysis. GC-FID analysis was carried out on a capillary gas chromatograph equipped with a fused silica capillary column (60 m length, 0.25 mm i.d., 0.25 μm film thickness, polar phase) and an FID detector. The injector and detector were maintained at 250 °C. The oven temperature program was as follows: 80 °C (held for 2 min), ramp at 2.5 °C/min to 230 °C, hold for 6 min. Helium was used as the carrier gas (flow ~1 mL/min, with a split ratio ~50:1). Identification of individual fatty acids was accomplished by comparing the retention times of peaks with those of a 37-component FAME standard mix run under the same conditions. Fatty acid methyl ester peaks were quantified as the weight percent of total fatty acids using internal normalization. The GC method followed the AOCS Official Method Ce 1h-05/AOCS 966.06 for fatty acid analysis with minor modifications to adapt to the available instrumentation [25,26].

### 2.7. Tocopherol Analysis

The total tocopherol content of the oils was analyzed using high-performance liquid chromatography with fluorescence detection (HPLC-FLD). Total tocopherols were analyzed by HPLC-FLD on a Shimadzu Nexera X2 HPLC system with a silica column (250 mm × 4.6 mm, 5 μm). Excitation/emission wavelengths were set at 290/330 nm. α-Tocopherol standards (Sigma–Aldrich, St. Louis, MO, USA) were used for calibration. Oil samples were diluted in n-hexane (approximately 0.1 g oil in 10 mL hexane) and filtered through a 0.45 μm PTFE syringe filter. An aliquot (20 μL) of each diluted oil sample was injected into an HPLC system equipped with a normal-phase silica column (250 mm × 4.6 mm, 5 μm) and a fluorescence detector. The mobile phase was n-hexane with 0.5% isopropanol at a flow rate of ~1 mL/min under isocratic conditions. The detector was set to an excitation wavelength of 290 nm and an emission wavelength of 330 nm, which are suitable for tocopherol analysis.

### 2.8. Total Phenolic Content

The total phenolic content (TPC) of the seed oils was measured using the Folin–Ciocalteu method with methanolic oil extracts. Absorbance at 765 nm was measured with a UV-1800 spectrophotometer (Shimadzu, Kyoto, Japan). The results were expressed as mg of gallic acid equivalents (GAE) per 100 g of oil.

### 2.9. Antioxidant Activity (DPPH Assay)

The antioxidant capacity of the oils was evaluated using the DPPH free radical scavenging assay. A stable DPPH (2,2-diphenyl-1-picrylhydrazyl) radical solution was prepared in ethanol (approximately 0.1 mM, deep violet color). In a test tube, 100 μL of oil (diluted to a 1:10 ratio in ethanol to enhance miscibility) or oil extract was added to 3.9 mL of the DPPH solution. The mixture was vortexed and then incubated in the dark for 30 min at room temperature. The decrease in absorbance at 517 nm was measured using a spectrophotometer, against a blank of ethanol. The scavenging activity was calculated as the percentage of DPPH radical quenched, using the following formula: % inhibition = [(A_control − A_sample)/A_control] × 100, where A_control is the absorbance of the DPPH solution without a sample, and A_sample is the absorbance with the oil sample. A calibration curve using Trolox was also prepared (0–100 μM Trolox) to express the antioxidant activity in Trolox equivalent antioxidant capacity (TEAC). The results were reported as μmol of Trolox equivalents per gram of oil (μmol TE/g), as well as the percentage of DPPH inhibition at a fixed oil concentration. All measurements were taken in triplicate. The DPPH assay is a rapid and sensitive method for evaluating free radical scavenging efficacy, based on the DPPH color change (from violet to yellow), as it is reduced by antioxidants.

### 2.10. Oxidative Stability (Rancimat Test)

The oxidative stability of the seed oils was determined using the Rancimat accelerated oxidation test. An 892 Rancimat apparatus (Rancimat software version 1.20; Metrohm, Herisau, Switzerland) was used, following the AOCS Official Method Cd 12b-92 protocol. Oil samples (~3 g each) were placed in the Rancimat reaction vessels and subjected to accelerated oxidation at a constant high temperature (110 °C). A continuous airflow (20 L/h) was bubbled through each oil sample, and the volatile oxidation products released were captured in distilled water, where their conductivity was measured over time. The time required for a sharp increase in conductivity (due to secondary oxidation products) is recorded as the induction time, also known as the oxidative stability index (OSI). This induction time (in hours) represents the resistance of the oil to oxidation: a longer induction time indicates higher oxidative stability. Each oil sample (fermented and control) was tested at least in duplicate. Induction times were determined automatically by the Rancimat software. Typical induction periods for these berry seed oils ranged from a few hours up to around 10 h under the test conditions. The Rancimat method provides a reliable accelerated measure of lipid oxidation stability, and the chosen conditions (110 °C, 20 L/h airflow) are standard for many vegetable oils. After Rancimat analysis, the apparatus was cleaned thoroughly to avoid any cross-contamination between samples.

### 2.11. Nutritional Composition Analysis

The proximate composition of berry seeds and press cakes was determined according to standard AOAC methods. The protein content was measured using the Kjeldahl method (AOAC Official Method 978.04–Nitrogen (Total) in Fertilizers, AOAC International: Rockville, MD, USA, 2019 using a Kjeltec 8400 analyzer (FOSS, Hillerød, Denmark), with a nitrogen-to-protein conversion factor of 6.25. The ash content was obtained by incinerating samples in a muffle furnace at 550 °C until a constant weight was reached. The total carbohydrates were calculated determining the difference after measuring the moisture, protein, fat, and ash. Reducing sugars were quantified spectrophotometrically using the phenol–sulfuric acid method (Dubois), with glucose as the calibration standard. Crude fiber was determined according to the Weende method (AOAC Official Method 962.09–Crude Fiber in Feeds: Weende Method, AOAC International: Rockville, MD, USA, 2019), which involves sequential acid and alkali digestion, followed by gravimetric quantification. The total energy value of the samples was calculated using Atwater conversion factors.

### 2.12. Statistical Analysis

All the experiments were carried out in triplicate. Data were analyzed using IBM SPSS Statistics 29. In the statistical processing of the data obtained from the analysis of the chemical composition of the fruits, the standard deviation was calculated and presented alongside the mean values. MS Excel (Redmond, WA, USA) and IBM SPSS Statistics (Armonk, NY, USA) software packages were used for statistical analysis. One-way analysis of variance (ANOVA), along with the post hoc Tukey’s HSD test, was employed for statistical analysis. Differences were considered significant at *p* < 0.05. The antioxidant activity was evaluated by using the DPPH assay.

Data on tocopherols (mg/kg), total phenolic content (mg GAE/100 g), DPPH radical scavenging activity (% inhibition), and oxidative stability (h) were averaged from three independent replicates and then standardized via Z-score normalization prior to analysis. A hierarchical clustering heatmap was generated using Euclidean distance and Ward’s linkage to group samples according to similarities in their biochemical profiles. This approach enabled the simultaneous comparison of fermented (F) and non-fermented (NF) seed oils across species and highlighted co-variation among the measured variables.

For dimensionality reduction and the visualization of overall variance, principal component analysis (PCA) was performed on the same normalized dataset. The first two principal components (PC1 and PC2) were extracted and plotted to display sample clustering (score plot) and variable contributions (loading plot). Score plots differentiated berry species and treatment conditions, while loading plots indicated the relative influence of tocopherols, phenolics, DPPH activity, and oxidative stability on the principal components. All analyses and visualizations were performed in Python 3.11 using the following libraries: scikit-learn 1.4, pandas 2.2, seaborn 0.13, and matplotlib 3.8.

## 3. Results

Berry seeds are recognized as valuable sources of oils and characterized by high levels of unsaturated fatty acids [27,28,29]. In addition to these essential polyunsaturated fatty acids, certain species, such as blackcurrant, are notable for containing γ-linolenic acid, while viburnum is distinguished by its high oleic acid content. Saturated fatty acids generally represent only a minor fraction of berry seed oils, making them nutritionally desirable. The following results present the fatty acid composition, oil yields, and biochemical characteristics of different berry seeds and their press cakes, highlighting the influence of fermentation and oil pressing:

The fatty acid composition of berry seed oils is presented in Table 1. Across all species, polyunsaturated fatty acids (PUFAs) were dominant, ranging from 65% to 80% of total fatty acids, with linoleic acid (C18:2, ω-6) and α-linolenic acid (C18:3, ω-3) being the major components. Raspberry oil contained approximately 45% linoleic acid and 29% α-linolenic acid, while strawberry oil contained 42% linoleic and 35% α-linolenic acid. Blackcurrant oil also contained γ-linolenic acid (GLA, 12–15%), in addition to linoleic (40–45%) and α-linolenic (12–15%). Viburnum oil was unique among the studied species, being dominated by oleic acid (>55%), while honeysuckle and rowanberry were characterized by high proportions of linoleic acid with negligible ω-3 content.

Fermentation did not significantly alter the relative fatty acid composition in any species (*p* > 0.05). Differences between NF and F samples were generally within 1–2 percentage points. Saturated fatty acids represented a minor fraction in all oils (<10%), with palmitic acid (C16:0) as the main contributor.

Oil pressing removed the majority of extractable lipids from the seeds, leaving press cakes with significantly reduced fat content. On average, the press cakes retained only a small residual oil fraction (generally a few percent of dry mass), indicating that 70–90% of the seed oil was recovered during pressing. Fermentation tended to further reduce the residual oil in press cakes by improving extraction: fermented samples often exhibited significantly decreased (*p* < 0.05) fat content in the press cakes (viburnum PC/F retained ~1% vs. ~2% in PC/NF), which is consistent with their higher pressing yields. Overall, oil pressing significantly reduced the fat content from pomace to press cake, demonstrating efficient oil recovery, especially in fermented treatments. This trend is clearly reflected in Table 1, which shows the sharp drop in total fat percentage from the whole seed (*p*) to the press cake (PC) for every berry species.

Despite large differences in oil quantity, the FA profiles of the oils were broadly similar across all berry species in that unsaturated fatty acids dominated. In the seed oils from non-fermented pomace, polyunsaturated fatty acids (PUFAs) were the predominant fraction in nearly all cases, comprising roughly 70–80% of the total FAs. Linoleic acid (C18:2, an omega-6 PUFA) was typically the single most abundant fatty acid, accompanied by appreciable levels of α-linolenic acid (C18:3, omega-3 PUFA) in several species.

Red currant seed oil had about 71% PUFA (primarily linoleic ~51% and α-linolenic ~20%). In contrast, honeysuckle and rowanberry showed PUFA profiles dominated almost entirely by linoleic acid with negligible omega-3 content. Honeysuckle seed oil consisted of ~71% linoleic acid and essentially 0% α-linolenic acid (no omega-3 detected), and rowanberry seed oil similarly contained ~67–70% linoleic and no measurable α-linolenic acid in our samples.

MUFAs, chiefly oleic acid (C18:1), made up the second-largest fraction in most berry oils. In seven of the eight species, oleic acid ranged from ~15% up to ~22% of total FAs. For instance, honeysuckle seed oil contained ~22% oleic acid, sea buckthorn contained ~19% oleic acid, red currant contained ~19% oleic acid, and rowanberry contained ~18% oleic acid. Strawberry, raspberry, and blackcurrant oils were on the lower end, with oleic contents of around 15–16%. Viburnum seed oil was a clear outlier: it was rich in MUFA, with oleic acid accounting for ~56–57% of total FAs in our analysis.

SFAs were only a minor component in all the berry seed oils. The total SFA levels generally made up less than 8–10% of the fatty acid pool, and in several cases, they made up a much lower percentage. Palmitic acid (C16:0) was the main saturated fatty acid detected, present at ~3–7% in most oils, with trace amounts of stearic acid (C18:0) and others making up the remainder of the saturates. Honeysuckle and rowanberry seed oils had the lowest SFA contents, with palmitic acid only making up ~1% of the total and total saturates likely making up around 2% of the total or less. These two oils were essentially composed almost entirely of unsaturated FAs (~98% unsaturated FAs). Among the surveyed berries, sea buckthorn had the highest saturated fat percentage, whereas honeysuckle and rowan had the lowest. Overall, all berry seed oils can be characterized as highly unsaturated, with PUFA as the dominant fraction (except in viburnum, where MUFA dominates) and with minimal saturated fat content.

Neither fermentation nor the mechanical pressing process caused any major changes to the fatty acid composition of the berry seed oils. Comparing non-fermented vs. fermented samples for each berry, the proportions of SFA, MUFA, and PUFA remained very similar. In every case, differences between P/NF and P/F in the percentage of each fatty acid class were within 1–2 percentage points (often within analytical error). These minimal differences indicate that fermentation treatment did not appreciably alter the fatty acid biosynthesis or composition of the seeds. In particular, the balance between linoleic and α-linolenic acids in each species remained constant, suggesting no selective degradation or production of omega-3 vs. omega-6 during fermentation. The fermented press cakes likewise showed fatty acid profiles nearly identical to those of the non-fermented press cakes, confirming that fermentation did not affect the residual oil composition. Any small numeric differences in FA percentages between NF and F samples were not statistically significant and likely stemmed from natural sample variation rather than a true fermentation effect.

Cold pressing extracted all fatty acid classes proportionally, leaving residual oils in press cakes with similar profiles to the original oils. Therefore, we can conclude that the fatty acid composition of these berry seed oils is an inherent trait of the species and is largely robust to the post-harvest treatments applied (fermentation and mechanical pressing). The press cakes’ residual oils retained the high-unsaturation characteristics of the original oils. For example, argan seed press cake oil has been shown to remain high in oleic (~48%) and linoleic (~32%) acids in the literature, and analogously in our study, each berry’s press cake oil composition mirrored that of the initial oil.

Table 2 presents the proximate nutritional composition of various berry seed samples under different treatment conditions. These results allow us to compare how fermentation alters the nutritional profile of the seed materials and how the press cake changes the composition relative to the whole seed powder.

Across all the berry types, fermentation had a marked impact on certain nutrients. In the whole seed, fermentation led to a significant increase in the protein content alongside a sharp decrease in total carbohydrates and sugars. This trend is clearly illustrated by blackcurrant seeds, where protein rose from about 13.4% in the unfermented powder to 20.0% after fermentation, while sugars dropped from 3.19% to just 0.54%. These changes suggest that the microorganisms used in fermentation consumed a portion of the available carbohydrates (particularly free sugars) and likely converted them into microbial biomass, which enhances the relative protein content of the material.

Consequently, the fermented seed powders show significantly lower carbohydrate and caloric values compared to their non-fermented counterparts. The reduction in energy content after fermentation is modest but consistent. Fermented blackcurrant seed powder has about 450 kcal/100 g, down from 461 kcal in the non-fermented sample, reflecting the loss of fermentable energy-rich components. Fiber content, on the other hand, remained relatively stable through fermentation, indicating that the structural polysaccharides largely were not broken down by the fermentation process in these short-term treatments. Ash content also showed minimal change with fermentation, except in a few cases were it significantly increased (*p* < 0.05), possibly due to concentration effects as other constituents were metabolized. Overall, fermentation primarily depleted the seeds’ available carbohydrates (especially sugars), thereby enriching the remaining seed matrix in protein and minerals, while leaving most of the fiber intact.

A comparison between the whole seed powders and their corresponding defatted press cakes reveals substantial differences in composition. The removal of oil from seeds leads to a dramatic reduction in total energy content and a relative enrichment of fiber. Non-fermented press cakes are extremely rich in dietary fiber for nearly all berry types, much higher than in the respective whole seed powders. Blackcurrant PC/NF contains about 59.8 g fiber per 100 g, compared to 18.6 g in the blackcurrant P/NF. This is because the oily fraction, which contributed substantial calories but no fiber, has been extracted, leaving behind a matrix concentrated in fibrous seed coats and cell wall materials. Accordingly, the caloric value of the press cakes plummets to around one-third or less of the whole seed value. Blackcurrant press cakes provide only ~155 kcal/100 g (649 kJ), versus ~461 kcal (1986 kJ) for the whole seed powder. Similar trends are observed for the other seeds. These differences confirm that most of the high-energy oil has been removed in the press cake, leaving a low-fat, high-fiber residue. The press cakes also tend to show moderately higher ash content (minerals) in some cases, likely because minerals remain with the solid fraction during oil pressing.

The protein content of press cakes generally falls within a similar range or is significantly increased (*p* < 0.05) compared to the whole seeds on a percentage basis, but the effect varies by berry. Strawberry seed press cakes contain about 10.9–11.0% protein, a bit higher than the 8.5–10.0% in the whole seed powder, which meets the expectation that removing oil concentrates the other constituents (protein, fiber, and minerals) in the remaining mass.

When the press cakes were fermented, the magnitude of changes was more subtle than in the whole seed powders, but the same general direction was observed. Because the press cakes contain very little simple carbohydrates to begin with, there is less fermentable substrate available, resulting in smaller changes. The protein content of the press cakes showed minimal change with fermentation. In most cases, the differences between PC/NF and PC/F protein were not statistically significant (e.g., blackcurrant PC/NF 14.03% vs. PC/F 14.12%; strawberry 10.87% vs. 11.02%). This is expected, since without much available carbon energy source in the press cake, there is limited new microbial biomass production. Similarly, fiber content remained high and essentially unchanged in PCs after fermentation (around 60% in all cases, with any small variations falling within experimental error).

In addition to examining the seed composition, we evaluated the cold-pressed oils obtained from fermented and non-fermented berry seeds. Table 3 presents a comparative profile of these oils, including the extracted oil yield, major fatty acid fractions (palmitic, oleic, and ω6 and ω3 polyunsaturated fatty acids), as well as key bioactive components (tocopherols and total phenolic content) and indicators of antioxidant activity (DPPH radical scavenging) and oxidative stability. This table allows us to assess how the fermentation pretreatment influenced the quantity and quality of the berry seed oils for each berry type, side by side.

As shown in Table 3, fermentation had a minimal impact on the fatty acid composition of the berry seed oils. For all berry types, the levels of palmitic acid (C16:0) and oleic acid (ω9), as well as the proportions of ω6 and ω3 polyunsaturated fatty acids, remained very similar between oils from non-fermented and fermented seeds. Each berry species maintained its characteristic oil profile regardless of fermentation. Viburnum seed oil remained rich in oleic acid (over fifty-five percent), whereas strawberry and seabuckthorn seed oils retained their high ω3 PUFA content (around one-third of total fatty acids) in both NF and F samples. This consistency indicates that the fermentation process did not substantially alter the fundamental lipid makeup of the oils.

In contrast, oil yield was somewhat affected by fermentation in a species-dependent manner. Blackcurrant and honeysuckle seeds showed a significant increase (*p* < 0.05) in oil yield after fermentation (e.g., blackcurrant oil yield rose from about 14% in NF to ~15% in F seeds). This suggests that the fermentation step may have improved oil release or extractability in these cases, possibly by softening the seed matrix or reducing viscosity. On the other hand, raspberry and viburnum seeds yielded significantly less oil when fermented (for example, raspberry oil yield decreased from ~9.0% to ~8.0%). The reduced yield in these berries could be due to minor losses or consumption of oil during fermentation or changes in seed texture that made oil extraction less efficient. Strawberry, redcurrant, sea buckthorn, and rowanberry seeds exhibited virtually no change in oil yield between NF and F samples, indicating that fermentation did not markedly influence the extractable oil quantity for those seeds.

The bioactive compounds in the oils and their antioxidant capacity showed only modest differences between NF and F seeds. Fermented seed oils tended to have slightly lower tocopherol levels and total phenolic contents compared to their non-fermented counterparts, although the changes were not dramatic. For instance, the total phenolics in strawberry seed oil dropped from roughly 11.0 to 10.0 mg GAE/100 g after fermentation, and tocopherols in blackcurrant seed oil remained high (around 1700 mg/kg), with only a minor reduction in the fermented sample.

Correspondingly, the DPPH scavenging activity of the oils, which reflects their antioxidant power, was only marginally reduced by fermentation. In most cases, the DPPH inhibition percentage decreased by just a few points (e.g., blackcurrant oil went from ~85% inhibition in NF to ~82% in F, and similar small declines were observed for other berries). These small but statistically significant (*p* < 0.05) decreases in antioxidants had a consistent but minor effect on oxidative stability: oils from fermented seeds showed a somewhat shorter induction period (stability time) compared with those from non-fermented seeds. The oxidative stability of blackcurrant seed oil was about 6.0 h for F seeds versus 6.5 h for NF seeds, indicating a small reduction in shelf-life stability upon fermentation. A similar trend was noted across other oils (fermented rowanberry seed oil exhibited ~2.8 h stability vs. ~3.0 h in NF). From a practical perspective, fermentation can be applied to berry seeds without significantly compromising the oil’s nutritional and oxidative quality. It may even improve oil yields for certain berries, although in others, a significant decrease in yield might occur. These findings suggest that solid-state fermentation is a generally feasible pre-treatment in the production of cold-pressed berry seed oils, maintaining most of the beneficial oil characteristics while potentially offering other processing benefits.

To comprehensively examine the antioxidant landscape of berry seed oils, we applied a multivariate clustering approach using heatmap analysis based on Z-score normalized data. This integrative visualization enabled the simultaneous assessment of tocopherols, total phenolics, DPPH radical scavenging capacity, and oxidative stability across all samples. By condensing the dataset into clustered biochemical signatures, the method highlights species-specific antioxidant profiles and reveals how closely fermented and non-fermented oils align within each group. This approach provides an intuitive overview of complex interactions between nutritional traits, emphasizing underlying patterns that may not be apparent from univariate comparisons alone.

The clustering pattern emphasizes that species identity was the main determinant of oil biochemical properties, with blackcurrant and red currant forming a group characterized by high tocopherols and phenolic content, while viburnum clustered separately due to its high oleic acid content and longer oxidative stability. Honeysuckle and rowanberry, with very low ω-3 PUFA levels, were grouped at the opposite end of the heatmap with relatively low antioxidant markers. Fermentation produced only minor shifts within each cluster, underlining that seed oil quality traits are largely species-specific rather than treatment-dependent. These observations confirm the robustness of berry seed oils as nutritionally valuable products even after pomace fermentation, aligning with patterns observed in olive byproducts where cultivar and tissue type explained more variance than processing treatment.

**Figure 1:**
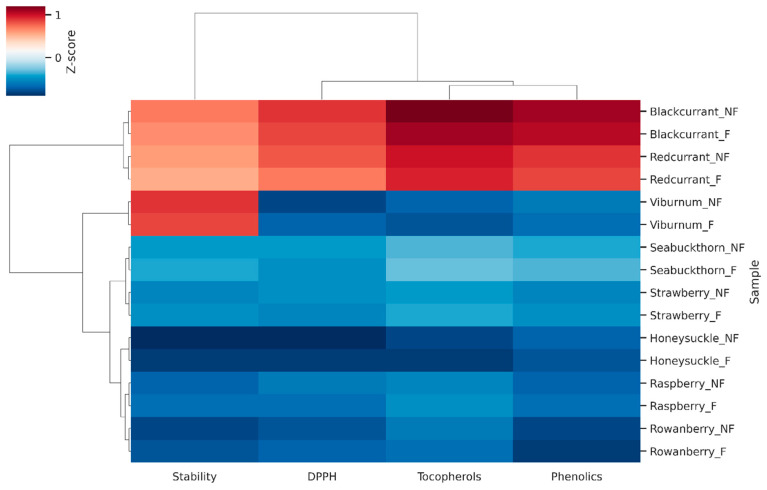
Heatmap of tocopherols, total phenolics, DPPH, and oxidative stability (Z-score normalized values) in berry seed oils. **Note:** Hierarchical clustering of NF and F seed oils from eight berry species. Data represent Z-score normalized values for tocopherols (mg/kg), total phenolics (mg GAE/100 g), DPPH radical scavenging activity (% inhibition), and oxidative stability (h). Higher values are shown in red, and lower values are shown in blue. Clusters highlight species-specific groupings, with fermentation producing only minor within-species shifts.

**Figure 2:**
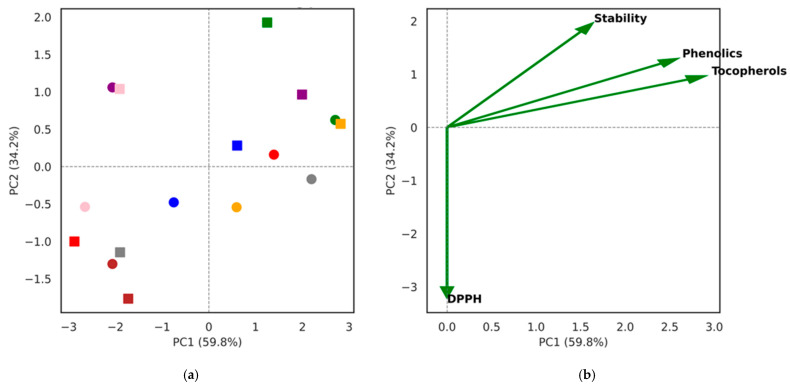
Principal component analysis (PCA) of antioxidant and stability parameters in berry seed oils. Note: (**a**) PCA score plot of NF, circles and F, squares seed oils from eight berry species: BC/blue (blackcurrant), S/orange (strawberry), RC/green (red currant), R/red (raspberry), SB/purple (sea buckthorn), H/brown (honeysuckle), V/pink (viburnum), and RW/gray (rowanberry). PC1 (59.8%) and PC2 (34.2%) explain over 94% of the variance. (**b**) PCA loading plot showing variable contributions: tocopherols, phenolics, and DPPH load strongly on PC1, while oxidative stability loads on PC2. Species identity is the main driver of clustering, while fermentation introduces only minor within-species variation.

The multivariate heatmap and PCA analyses (Figure 1 and Figure 2) confirmed that species identity was the dominant factor influencing biochemical variation, while fermentation introduced only minor within-species shifts. Clustering grouped blackcurrant and red currant together (high tocopherols and phenolics), viburnum separately (oleic acid–rich), and honeysuckle/rowanberry at the opposite end (low ω-3 PUFA and antioxidant markers). Fermentation effects were minor, and species remained closely paired across treatments.

The PCA clearly demonstrated that species identity rather than fermentation status was the main determinant of oil characteristics. Blackcurrant and red currant clustered together, reflecting their high tocopherol and phenolic contents that aligned with strong DPPH scavenging activity. In contrast, viburnum was separated along PC2, driven by its higher oxidative stability associated with elevated oleic acid levels, while honeysuckle and rowanberry were grouped at the opposite end with lower antioxidant marker loadings. The close positioning of non-fermented and fermented samples within each berry species indicated that fermentation introduced only minor shifts in antioxidant and stability profiles compared to inherent inter-species differences. Variable loadings confirmed that tocopherols, phenolics, and DPPH were strongly correlated, jointly influencing PC1, while oxidative stability predominantly contributed to PC2. Together, these results reinforce the finding that berry seed oils retain their distinctive biochemical signatures after fermentation, with antioxidant capacity being largely dependent on natural tocopherol and phenolic abundance and stability governed by fatty acid composition.

## 4. Discussion

The present study demonstrates that primary fermentation exerts only moderate effects on the nutritional composition of berry seeds and the quality of their cold-pressed oils, although the direction and magnitude of these changes are highly species-dependent. In general, fermentation resulted in a relative increase in seed protein and a marked decrease in simple carbohydrates and sugars, which is consistent with the microbial utilization of readily available substrates. These observations are in agreement with previous reports showing that the solid-state fermentation of plant residues enhances protein content through microbial biomass production while reducing carbohydrate fractions [30,31]. The preservation of dietary fiber across all species suggests that structural polysaccharides are largely resistant to the short fermentation period, a finding that echoes studies on cereal bran and fruit pomaces [32,33].

The observation that oil yield was increased in certain species (e.g., blackcurrant and honeysuckle) but statistically significantly reduced in others (e.g., raspberry and viburnum) highlights the importance of seed microstructure and fermentation conditions. Similar variability has been reported in grape pomace, where fermentation either facilitated or impeded oil extraction depending on matrix composition and fermentation intensity [34]. The general stability of fatty acid composition across fermented and non-fermented samples supports the hypothesis that primary fermentation does not induce major lipid metabolism within intact seeds, aligning with reports from both grape and flaxseed systems [35,36]. This stability is particularly relevant for industrial production, as it ensures the preservation of nutritional quality dominated by linoleic, α-linolenic, and γ-linolenic acids, as well as oleic acid in viburnum [37,38].

Fermentation did not cause statistically significant changes in tocopherol or total phenolic contents. Antioxidant activity and oxidative stability were also unaffected. These results indicate that fermentation did not compromise the nutritional or functional quality of the oils processed in this way [39,40]. Nevertheless, the reductions were minor, and the oils retained strong antioxidant capacity comparable to values reported in the literature for cold-pressed berry oils [41]. The mechanism behind these reductions may be linked to the leaching of water-soluble phenolics into the fermenting medium, enzymatic degradation by yeasts or bacteria, and increased oxidative stress due to ethanol and oxygen exposure during fermentation [39,42]. Our results confirmed that tocopherol levels remained within a statistically non-significant range between non-fermented and fermented samples, supporting their nutritional relevance.

The broad context of these findings supports the valorization of berry processing byproducts. Fermentation is often unavoidable in fruit wine or juice production, and concerns have been raised regarding whether fermented pomace is inferior to fresh seed material for oil recovery. Our comparative approach across eight berry species provides strong evidence that fermented seeds can yield oils of comparable fatty acid quality and only slightly reduced antioxidant potential, echoing previous findings from grape seed oil extracted after winemaking [43,44]. The demonstration across eight berry species adds novelty, as previous studies typically focused on single species or grape pomace.

Fermentation-driven compositional changes in berry seeds can be better understood by drawing on parallel findings in other oil-rich matrices. For example, studies on soybean and sunflower have shown that microbial metabolism primarily targets soluble carbohydrates, while the lipid fraction remains largely unaffected [45,46]. This supports our observation that fatty acid profiles were conserved across treatments, despite marked reductions in seed sugars. Such evidence indicates that the lipid fraction in berry seeds is structurally resilient to short-term microbial activity, reinforcing its stability for downstream cold-pressing applications. Beyond compositional stability, fermentation may also influence the technological valorization of seeds. Investigations in olive pomace and pomegranate residues have demonstrated that controlled microbial activity can enhance oil extractability by weakening cell wall structures [47,48]. Our finding that oil yield increased significantly in some species, such as blackcurrant and honeysuckle, is consistent with this mechanism. The implication is that fermentation, rather than being a drawback, can act as a pre-treatment step that improves oil recovery while maintaining nutritional quality under specific conditions.

Some limitations of the present study must be acknowledged. The fermentation conditions were standardized (21 °C, 14 days, with added water and sugar), but industrial processes vary considerably and may produce different outcomes. Only one yeast strain was employed; mixed microbial consortia, including lactic acid bacteria, could alter the metabolic profile of seeds and oils. In addition, only selected compositional and antioxidant parameters were assessed; other bioactive constituents, such as sterols or carotenoids, were not measured and may respond differently to fermentation. Oxidative stability was evaluated under accelerated conditions, which may not fully predict shelf life under commercial storage. Future research should investigate the influence of different microbial consortia, fermentation regimes, and process intensities and extend analyses to additional bioactives such as sterols or carotenoids. Multi-omics approaches could help investigate microbial–seed interactions and their consequences for lipid and phenolic stability. Furthermore, sensory evaluations and application trials in food, cosmetics, and nutraceuticals are warranted to establish consumer acceptance and industrial potential of oils from fermented seeds. This study confirms that species identity is the dominant driver of oil biochemical composition, while fermentation introduces only minor within-species shifts.

## 5. Conclusions

This study provides the first systematic comparison of oils obtained from fermented and non-fermented seeds across eight berry species. Despite species-specific variation in oil yield, fermentation did not significantly alter fatty acid composition, ensuring the preservation of essential nutritional traits, such as high levels of linoleic and α-linolenic acids.

The innovative contribution of this work lies in showing that seeds remaining after juice making and primary fermentation can still be valorized for cold-pressed oil production with minimal compromise in quality. This finding addresses a critical gap in byproduct utilization and demonstrates that fermentation, rather than diminishing value, can facilitate oil recovery under certain conditions.

From a practical perspective, these results highlight opportunities for sustainable processing in the food, cosmetic, and nutraceutical sectors. The valorization of fermented berry pomace as a raw material for oil extraction supports circular bioeconomy principles and provides a scalable pathway to reduce waste in berry processing chains.

## Figures and Tables

**Table 1:** Fat composition of fermented vs. non-fermented berry seeds.

Berry	State	Fat (%)	Saturated FAs (%)	Monounsaturated FAs (%)	Polyunsaturated FAs (%)
**Blackcurrant**	P/NF	20.23 ± 0.28 ^a^	1.71 ± 0.06 ^a^	3.52 ± 0.09 ^a^	15.83 ± 0.22 ^a^
	P/F	21.04 ± 0.31 ^a^	1.73 ± 0.05 ^a^	3.55 ± 0.08 ^a^	16.02 ± 0.25 ^a^
	PC/NF	1.82 ± 0.11 ^b^	0.21 ± 0.02 ^b^	0.33 ± 0.02 ^b^	1.28 ± 0.06 ^b^
	PC/F	1.95 ± 0.12 ^b^	0.22 ± 0.02 ^b^	0.31 ± 0.02 ^b^	1.42 ± 0.07 ^b^
**Strawberry**	P/NF	8.64 ± 0.15 ^a^	0.79 ± 0.05 ^a^	1.68 ± 0.07 ^a^	5.95 ± 0.14 ^a^
	P/F	8.75 ± 0.16 ^a^	0.81 ± 0.05 ^a^	1.63 ± 0.06 ^a^	5.91 ± 0.13 ^a^
	PC/NF	0.97 ± 0.08 ^b^	0.08 ± 0.01 ^b^	0.19 ± 0.02 ^b^	0.70 ± 0.04 ^b^
	PC/F	1.04 ± 0.09 ^b^	0.09 ± 0.01 ^b^	0.20 ± 0.02 ^b^	0.75 ± 0.05 ^b^
**Red currant**	P/NF	10.68 ± 0.27 ^a^	1.28 ± 0.06 ^a^	3.88 ± 0.09 ^a^	14.67 ± 0.24 ^a^
	P/F	10.97 ± 0.28 ^a^	1.31 ± 0.05 ^a^	3.95 ± 0.08 ^a^	14.88 ± 0.25 ^a^
	PC/NF	1.03 ± 0.09 ^b^	0.09 ± 0.01 ^b^	0.19 ± 0.02 ^b^	0.75 ± 0.05 ^b^
	PC/F	1.08 ± 0.10 ^b^	0.10 ± 0.01 ^b^	0.21 ± 0.02 ^b^	0.77 ± 0.05 ^b^
**Raspberry**	P/NF	16.57 ± 0.34 ^a^	1.59 ± 0.06 ^a^	3.09 ± 0.08 ^a^	17.12 ± 0.29 ^a^
	P/F	21.61 ± 0.35 ^a^	1.61 ± 0.05 ^a^	3.11 ± 0.07 ^a^	16.95 ± 0.28 ^a^
	PC/NF	1.11 ± 0.09 ^b^	0.05 ± 0.01 ^b^	0.16 ± 0.02 ^b^	0.90 ± 0.05 ^b^
	PC/F	1.07 ± 0.08 ^b^	0.05 ± 0.01 ^b^	0.14 ± 0.01 ^b^	0.82 ± 0.04 ^b^
**Sea** **buckthorn**	P/NF	18.21 ± 0.26 ^a^	1.88 ± 0.07 ^a^	5.09 ± 0.14 ^a^	10.54 ± 0.27 ^a^
	P/F	18.25 ± 0.27 ^a^	1.91 ± 0.06 ^a^	5.07 ± 0.13 ^a^	10.47 ± 0.25 ^a^
	PC/NF	1.23 ± 0.10 ^b^	0.12 ± 0.01 ^b^	0.23 ± 0.02 ^b^	0.88 ± 0.05 ^b^
	PC/F	1.19 ± 0.09 ^b^	0.11 ± 0.01 ^b^	0.22 ± 0.02 ^b^	0.86 ± 0.04 ^b^
**Honeysuckle**	P/NF	15.14 ± 0.23 ^a^	0.61 ± 0.04 ^a^	1.47 ± 0.06 ^a^	11.31 ± 0.29 ^a^
	P/F	15.09 ± 0.24 ^a^	0.63 ± 0.04 ^a^	1.45 ± 0.05 ^a^	11.28 ± 0.27 ^a^
	PC/NF	0.83 ± 0.07 ^b^	0.05 ± 0.01 ^b^	0.14 ± 0.01 ^b^	0.51 ± 0.03 ^b^
	PC/F	0.91 ± 0.08 ^b^	0.06 ± 0.01 ^b^	0.15 ± 0.01 ^b^	0.61 ± 0.04 ^b^
**Viburnum**	P/NF	6.72 ± 0.18 ^a^	0.32 ± 0.02 ^a^	3.81 ± 0.09 ^a^	2.59 ± 0.11 ^a^
	P/F	5.91 ± 0.16 ^a^	0.29 ± 0.02 ^a^	3.27 ± 0.08 ^a^	2.35 ± 0.10 ^a^
	PC/NF	0.73 ± 0.07 ^b^	0.03 ± 0.01 ^b^	0.27 ± 0.02 ^b^	0.16 ± 0.02 ^b^
	PC/F	0.69 ± 0.06 ^b^	0.03 ± 0.01 ^b^	0.25 ± 0.02 ^b^	0.15 ± 0.02 ^b^
**Rowanberry**	P/NF	11.94 ± 0.21 ^a^	1.03 ± 0.05 ^a^	2.54 ± 0.07 ^a^	8.37 ± 0.22 ^a^
	P/F	11.88 ± 0.19 ^a^	1.06 ± 0.04 ^a^	2.48 ± 0.06 ^a^	8.34 ± 0.20 ^a^
	PC/NF	1.08 ± 0.09 ^b^	0.09 ± 0.01 ^b^	0.22 ± 0.02 ^b^	0.77 ± 0.04 ^b^
	PC/F	1.02 ± 0.08 ^b^	0.08 ± 0.01 ^b^	0.21 ± 0.02 ^b^	0.73 ± 0.03 ^b^

**Note:** Values represent mean ± SD of three replicates. Different superscript letters (a,b) in the same column indicate significant differences (*p* < 0.05). P/NF = pomace (after juice) non-fermented; P/F = pomace fermented; PC/NF = press cake from non-fermented seeds; PC/F = press cake from fermented seeds.

**Table 2:** Nutritional composition of F vs. NF berry seeds.

Berry	Sample	Protein (%)	Carbohydrates (%)	Sugars (%)	Fiber (g)	Ash (%)	Energy (kcal)	Energy (kJ)
**Blackcurrant**	P/NF	13.42 ± 0.33 ^a^	54.07 ± 0.48 ^a^	3.19 ± 0.07 ^a^	18.62 ± 0.31 ^a^	2.81 ± 0.07 ^a^	460.75 ± 2.35 ^a^	1986.23 ± 6.18 ^a^
	P/F	19.98 ± 0.42 ^b^	39.85 ± 0.51 ^b^	0.54 ± 0.03 ^b^	18.07 ± 0.39 ^a^	3.46 ± 0.08 ^b^	449.88 ± 2.12 ^b^	1879.54 ± 5.92 ^b^
	PC/NF	14.03 ± 0.29 ^a^	20.75 ± 0.62 ^b^	10.36 ± 0.34 ^c^	59.84 ± 0.83 ^b^	3.52 ± 0.09 ^b^	155.42 ± 3.16 ^c^	649.32 ± 8.42 ^c^
	PC/F	14.12 ± 0.27 ^a^	20.38 ± 0.59 ^b^	2.04 ± 0.09 ^b^	60.12 ± 0.76 ^b^	3.47 ± 0.08 ^b^	156.18 ± 3.24 ^c^	652.47 ± 8.35 ^c^
**Strawberry**	P/NF	8.46 ± 0.21 ^a^	65.72 ± 0.53 ^a^	5.28 ± 0.12 ^a^	10.12 ± 0.28 ^a^	3.91 ± 0.09 ^a^	374.32 ± 2.45 ^a^	1611.72 ± 6.24 ^a^
	P/F	9.97 ± 0.26 ^b^	61.98 ± 0.56 ^b^	2.05 ± 0.10 ^b^	9.94 ± 0.25 ^a^	3.82 ± 0.08 ^a^	362.15 ± 2.36 ^b^	1515.33 ± 6.02 ^b^
	PC/NF	10.87 ± 0.31 ^a^	25.36 ± 0.64 ^b^	12.51 ± 0.38 ^c^	59.82 ± 0.82 ^b^	2.71 ± 0.07 ^a^	143.65 ± 2.92 ^c^	600.54 ± 8.16 ^c^
	PC/F	11.02 ± 0.28 ^a^	25.08 ± 0.61 ^b^	2.43 ± 0.11 ^b^	60.15 ± 0.79 ^b^	2.69 ± 0.07 ^a^	144.22 ± 2.95 ^c^	603.18 ± 8.09 ^c^
**Red currant**	P/NF	16.71 ± 0.38 ^a^	52.36 ± 0.48 ^a^	1.21 ± 0.06 ^a^	20.92 ± 0.43 ^a^	2.23 ± 0.06 ^a^	462.45 ± 2.62 ^a^	1931.84 ± 6.42 ^a^
	P/F	18.04 ± 0.41 ^b^	49.92 ± 0.51 ^b^	0.47 ± 0.04 ^b^	20.54 ± 0.46 ^a^	2.14 ± 0.05 ^a^	449.87 ± 2.44 ^b^	1879.64 ± 6.21 ^b^
	PC/NF	12.97 ± 0.29 ^a^	22.73 ± 0.63 ^b^	11.42 ± 0.35 ^c^	59.97 ± 0.84 ^b^	3.51 ± 0.08 ^b^	153.27 ± 3.05 ^c^	640.12 ± 8.21 ^c^
	PC/F	13.12 ± 0.27 ^c^	22.42 ± 0.60 ^b^	2.26 ± 0.12 ^b^	60.08 ± 0.79 ^b^	3.48 ± 0.08 ^b^	152.86 ± 3.08 ^c^	639.27 ± 8.14 ^c^
**Raspberry**	P/NF	4.03 ± 0.12 ^c^	65.13 ± 0.54 ^a^	1.49 ± 0.07 ^a^	48.93 ± 0.47 ^a^	0.81 ± 0.04 ^a^	471.23 ± 2.64 ^a^	2030.15 ± 6.52 ^a^
	P/F	5.98 ± 0.15 ^b^	61.87 ± 0.52 ^b^	0.19 ± 0.03 ^b^	47.98 ± 0.49 ^a^	0.79 ± 0.04 ^a^	459.27 ± 2.42 ^b^	1979.32 ± 6.37 ^b^
	PC/NF	10.95 ± 0.26 ^a^	27.98 ± 0.62 ^b^	12.74 ± 0.38 ^c^	59.74 ± 0.82 ^b^	3.02 ± 0.07 ^b^	144.23 ± 2.91 ^c^	603.14 ± 8.16 ^c^
	PC/F	10.91 ± 0.25 ^c^	28.35 ± 0.65 ^b^	2.76 ± 0.11 ^b^	60.02 ± 0.79 ^b^	3.01 ± 0.07 ^b^	142.98 ± 2.89 ^c^	598.62 ± 8.04 ^c^
**Sea buckthorn**	P/NF	28.14 ± 0.55 ^c^	42.53 ± 0.49 ^a^	1.49 ± 0.06 ^a^	16.92 ± 0.43 ^a^	2.29 ± 0.06 ^a^	445.83 ± 2.58 ^a^	1920.61 ± 6.41 ^a^
	P/F	28.96 ± 0.52 ^a^	41.03 ± 0.46 ^b^	0.23 ± 0.03 ^b^	16.49 ± 0.41 ^a^	2.32 ± 0.05 ^a^	438.27 ± 2.33 ^b^	1834.62 ± 6.22 ^b^
	PC/NF	13.34 ± 0.31 ^a^	22.72 ± 0.59 ^b^	11.26 ± 0.35 ^c^	59.92 ± 0.84 ^b^	3.01 ± 0.08 ^a^	159.63 ± 3.04 ^c^	669.32 ± 8.12 ^c^
	PC/F	13.25 ± 0.30 ^a^	22.68 ± 0.61 ^b^	2.27 ± 0.12 ^b^	59.84 ± 0.81 ^b^	3.02 ± 0.07 ^a^	158.47 ± 3.02 ^c^	664.75 ± 8.07 ^c^
**Honeysuckle**	P/NF	11.58 ± 0.33 ^a^	64.19 ± 0.47 ^a^	3.81 ± 0.11 ^a^	10.73 ± 0.29 ^a^	3.61 ± 0.08 ^a^	438.64 ± 2.48 ^a^	1889.23 ± 6.34 ^a^
	P/F	13.04 ± 0.36 ^b^	61.07 ± 0.50 ^b^	1.03 ± 0.05 ^b^	10.48 ± 0.27 ^a^	3.52 ± 0.07 ^a^	425.83 ± 2.32 ^b^	1780.31 ± 6.28 ^b^
	PC/NF	12.92 ± 0.34 ^a^	23.59 ± 0.61 ^b^	11.02 ± 0.34 ^c^	59.96 ± 0.85 ^b^	2.81 ± 0.07 ^a^	148.75 ± 2.87 ^c^	622.14 ± 7.98 ^c^
	PC/F	12.98 ± 0.33 ^a^	23.25 ± 0.63 ^b^	2.25 ± 0.11 ^b^	60.03 ± 0.82 ^b^	2.79 ± 0.07 ^a^	150.92 ± 2.90 ^c^	631.25 ± 8.05 ^c^
**Viburnum**	P/NF	10.02 ± 0.28 ^a^	24.38 ± 0.44 ^a^	12.19 ± 0.31 ^a^	56.37 ± 0.71 ^a^	2.47 ± 0.06 ^a^	197.84 ± 2.27 ^a^	827.46 ± 6.12 ^a^
	P/F	10.64 ± 0.30 ^b^	14.96 ± 0.41 ^b^	1.47 ± 0.07 ^b^	60.08 ± 0.74 ^a^	2.65 ± 0.07 ^a^	174.68 ± 2.11 ^b^	731.25 ± 6.08 ^b^
	PC/NF	10.55 ± 0.29 ^c^	26.04 ± 0.52 ^b^	12.62 ± 0.36 ^c^	59.93 ± 0.85 ^b^	2.86 ± 0.08 ^a^	141.68 ± 2.11 ^b^	724.31 ± 7.64 ^b^
**Rowanberry**	P/F	6.21 ± 0.18 ^c^	58.32 ± 0.54 ^a^	3.85 ± 0.12 ^a^	28.47 ± 0.51 ^a^	3.12 ± 0.07 ^a^	345.26 ± 2.42 ^a^	1443.86 ± 6.24 ^a^
	P/NF	7.15 ± 0.21 ^b^	55.14 ± 0.49 ^b^	0.96 ± 0.05 ^b^	28.83 ± 0.48 ^a^	3.25 ± 0.08 ^a^	331.47 ± 2.38 ^b^	1387.77 ± 6.12 ^b^
	PC/F	6.87 ± 0.19 ^a^	24.95 ± 0.63 ^b^	11.22 ± 0.35 ^c^	59.74 ± 0.82 ^b^	3.45 ± 0.09 ^b^	152.84 ± 2.97 ^c^	639.52 ± 8.15 ^c^
	PC/NF	6.94 ± 0.20 ^a^	24.63 ± 0.61 ^b^	2.21 ± 0.11 ^b^	60.02 ± 0.79 ^b^	3.42 ± 0.08 ^b^	151.23 ± 2.91 ^c^	632.78 ± 8.07 ^c^

**Note:** Values represent the mean ± SD of three replicates. Different superscript letters a, b, and c indicate significant differences (*p* < 0.05). Parameters measured: protein (%), carbohydrates (%), sugars (%), fiber (mg/100 g), ash (%), and calculated energy (kcal and kJ per 100 g dry weight).

**Table 3:** Comparative analytical profile of F and NF cold-pressed berry seed oils.

Berry	State	Oil Yield (%)	Palmitic (C16:0) [%]	Oleic (ω-9) [%]	ω-6 PUFA [%]	ω-3 PUFA [%]	Tocopherols [mg/kg]	Total Phenolics [mg GAE/100 g]	DPPH Scavenging [% Inhibition]	Oxidative Stability [h]
Blackcurrant	NF	13.97 ± 0.17 ^b^	5.48 ± 0.15 ^a^	14.80 ± 0.20 ^a^	59.95 ± 0.80 ^a^	12.68 ± 0.76 ^a^	1711.07 ± 17.51 ^a^	19.28 ± 0.46 ^a^	84.89 ± 1.19 ^a^	6.47 ± 0.32 ^a^
Blackcurrant	F	14.99 ± 0.27 ^a^	5.52 ± 0.11 ^a^	15.04 ± 0.10 ^a^	61.20 ± 1.62 ^a^	12.31 ± 0.85 ^a^	1699.03 ± 12.30 ^a^	18.19 ± 0.52 ^a^	81.77 ± 1.04 ^a^	6.01 ± 0.28 ^a^
Strawberry	NF	8.61 ± 0.18 ^a^	3.65 ± 0.08 ^a^	15.90 ± 0.35 ^a^	42.02 ± 1.24 ^a^	35.61 ± 1.38 ^a^	701.36 ± 8.51 ^a^	11.01 ± 0.41 ^a^	75.28 ± 0.78 ^a^	3.98 ± 0.13 ^a^
Strawberry	F	8.65 ± 0.16 ^a^	3.77 ± 0.14 ^a^	16.08 ± 0.32 ^a^	43.30 ± 1.13 ^a^	34.76 ± 0.81 ^a^	692.08 ± 12.37 ^a^	10.01 ± 0.38 ^a^	71.98 ± 0.72 ^a^	3.83 ± 0.12 ^a^
Redcurrant	NF	8.01 ± 0.27 ^a^	4.97 ± 0.13 ^a^	18.92 ± 0.26 ^a^	50.86 ± 1.27 ^a^	20.31 ± 0.90 ^a^	1437.82 ± 9.55 ^a^	14.70 ± 0.42 ^a^	80.14 ± 0.84 ^a^	5.57 ± 0.24 ^a^
Redcurrant	F	8.01 ± 0.27 ^a^	5.03 ± 0.05 ^a^	19.20 ± 0.20 ^a^	52.00 ± 1.06 ^a^	19.48 ± 0.82 ^a^	1434.09 ± 2.00 ^a^	13.94 ± 0.34 ^a^	78.19 ± 0.69 ^a^	5.27 ± 0.17 ^a^
Raspberry	NF	8.98 ± 0.18 ^a^	3.21 ± 0.08 ^a^	15.27 ± 0.28 ^a^	44.59 ± 1.26 ^a^	29.30 ± 0.82 ^a^	886.39 ± 6.84 ^a^	12.09 ± 0.38 ^a^	82.18 ± 0.88 ^a^	4.54 ± 0.30 ^a^
Raspberry	F	7.99 ± 0.23 ^b^	3.13 ± 0.10 ^a^	15.06 ± 0.30 ^a^	44.75 ± 0.93 ^a^	29.14 ± 0.94 ^a^	878.10 ± 6.29 ^a^	11.03 ± 0.48 ^a^	79.29 ± 1.18 ^a^	4.25 ± 0.15 ^a^
Seabuckthorn	NF	9.95 ± 0.20 ^a^	7.39 ± 0.11 ^a^	19.18 ± 0.20 ^a^	37.35 ± 0.87 ^a^	32.96 ± 0.96 ^a^	599.86 ± 5.35 ^a^	12.00 ± 0.32 ^a^	78.43 ± 0.92 ^a^	3.76 ± 0.15 ^a^
Seabuckthorn	F	9.95 ± 0.17 ^a^	7.43 ± 0.12 ^a^	18.48 ± 0.27 ^a^	38.02 ± 0.78 ^a^	32.48 ± 0.79 ^a^	589.80 ± 4.31 ^a^	10.99 ± 0.25 ^a^	75.36 ± 0.75 ^a^	3.62 ± 0.12 ^a^
Honeysuckle	NF	7.01 ± 0.15 ^b^	0.99 ± 0.01 ^a^	22.05 ± 0.27 ^a^	71.34 ± 1.74 ^a^	0.00 ± 0.00 ^a^	204.03 ± 4.07 ^a^	8.97 ± 0.44 ^a^	89.88 ± 1.25 ^a^	2.48 ± 0.08 ^a^
Honeysuckle	F	7.98 ± 0.25 ^a^	1.02 ± 0.03 ^a^	22.05 ± 0.14 ^a^	70.52 ± 1.66 ^a^	0.00 ± 0.00 ^a^	199.37 ± 2.83 ^a^	8.97 ± 0.33 ^a^	87.80 ± 1.49 ^a^	2.26 ± 0.09 ^a^
Viburnum	NF	6.02 ± 0.22 ^a^	2.01 ± 0.03 ^a^	56.89 ± 0.44 ^a^	39.63 ± 0.70 ^a^	0.28 ± 0.10 ^a^	329.39 ± 4.40 ^a^	9.94 ± 0.34 ^a^	69.89 ± 0.68 ^a^	6.98 ± 0.28 ^a^
Viburnum	F	4.95 ± 0.19 ^b^	2.01 ± 0.03 ^a^	56.23 ± 0.58 ^a^	40.01 ± 0.45 ^a^	0.28 ± 0.07 ^a^	325.26 ± 1.55 ^a^	8.92 ± 0.47 ^a^	67.90 ± 0.61 ^a^	6.81 ± 0.21 ^a^
Rowanberry	NF	11.97 ± 0.19 ^a^	0.97 ± 0.05 ^a^	18.04 ± 0.33 ^a^	70.05 ± 1.53 ^a^	0.00 ± 0.00 ^a^	199.41 ± 3.17 ^a^	8.92 ± 0.22 ^a^	80.25 ± 0.85 ^a^	3.03 ± 0.20 ^a^
Rowanberry	F	11.97± 0.19 ^a^	0.97 ± 0.03 ^a^	18.04 ± 0.34 ^a^	66.86 ± 1.56 ^a^	0.00 ± 0.00 ^a^	194.27 ± 3.89 ^a^	8.41 ± 0.25 ^a^	77.84 ± 0.94 ^a^	2.78 ± 0.17 ^a^

**Note:** Values represent the mean ± SD of three replicates. Different superscript letters a and b in the same column indicate significant differences (*p* < 0.05). Parameters measured: oil yield (%), major fatty acids (palmitic, oleic, ω-6 PUFA, and ω-3 PUFA), bioactive compounds (tocopherols and total phenolics), antioxidant activity (DPPH scavenging %), and oxidative stability (h).

## Data Availability

Data are contained within the article.
